# Antiphospholipid syndrome: a case report with an unusual wide spectrum of clinical manifestations

**DOI:** 10.1186/s13317-019-0119-3

**Published:** 2019-10-19

**Authors:** Carmela Mazzoccoli, Domenico Comitangelo, Alessia D’Introno, Valeria Mastropierro, Carlo Sabbà, Antonio Perrone

**Affiliations:** Department of Interdisciplinary Medicine, University of Bari “Aldo Moro”, Piazza Giulio Cesare 11, 70124 Bari, Italy

**Keywords:** Antiphospholipid syndrome, Thrombocytopenia, Dizziness, Seizures, Non-bacterial thrombotic endocarditis, Acute pancreatitis

## Abstract

**Background:**

Antiphospholipid syndrome (APS) is an autoimmune disease characterized by the occurrence of venous and/or arterial thrombosis, and the detection of circulating antiphospholipid antibodies. The classification criteria for definite APS are actually met when at least one clinical criterion (thrombosis or pregnancy morbidity) is present in association of one laboratory criterion (LAC, aCL antibody or aβ2GPI antibody present on two or more occasions, at least 12 weeks a part), and thrombosis should be confirmed by objective validated criteria. The average age of primary APS patients has been reported to be about 35–40 years and the disease is more common in women than in men.

**Case presentation:**

In this report, we described a rare case of an adult male who presented over a period of 9 years with a wide spectrum of clinical manifestations involving different organs that were not initially diagnosed as APS. Dizziness and syncope were his first clinical symptoms, and a non-bacterial thrombotic endocarditis (NBTE) involving the mitral valve was at first diagnosed. Subsequently, the patient also presented with generalized seizures and subsequent head injury. When the patient was admitted to our clinic with bilateral epistaxis and fever, thrombocytopenia was revealed. Moreover, laboratory examinations showed acute pancreatitis with an increase of levels of inflammation markers.

**Conclusion:**

Based on the patient’s medical history and all the examination results, it was possible to make a diagnosis of primary APS and, starting from diagnosis of thrombocytopenia, we were allowed to conclude that all of manifestation were epi-phenomena of a unique clinical entity, rather than unrelated diseases. Though APS is one of the most common thrombocytophilias, unfortunately, it is not recognized often enough. The lack of prevention in undiagnosed patients may cause severe complications which can in turn result in the death of those patients.

## Background

Antiphospholipid syndrome (APS) is an autoimmune disorder characterized by a state of hypercoagulability potentially resulting in thrombosis of all segments of the vascular bed [1].

The syndrome is characterized clinically by recurrent venous and/or arterial thromboembolic events, or pregnancy morbidity. In addition to these clinical manifestations, the sine qua non for the syndrome is the persistent presence of a unique collection of autoantibodies that target specific phospholipid-binding proteins [2]. Antibodies against *β*2-glycoprotein I (aβ2GPI) and cardiolipin (aCL), together with the functional assay lupus anticoagulant (LAC), are the three laboratory tests considered in the revised criteria for the diagnosis of the syndrome [3].

Currently, we use the classification criteria for antiphospholipid syndrome (APS) formulated during the consensus conference in Sapporo and revised in Sydney [4].

The classification criteria for definite APS are actually met when at least one clinical criterion (thrombosis or pregnancy morbidity) is present in association of one laboratory criterion (LAC, aCL antibody or aβ2GPI antibody present on two or more occasions, at least 12 weeks a part), and thrombosis should be confirmed by objective validated criteria [3, 5].

However, there are several clinical manifestations not included in the classical revised criteria of APS such as thrombocytopenia, hemolytic anemia, cardiac valve disease, renal microangiopathy, livedo reticularis, neurologic disturbances, leg ulcers and amaurosis fugax [6] that are common features in APS patients and, as stated by Miyakis et al., they can be classified as “non-criteria features of APS”.

Prevalence of the aPL in the general population ranges between 1 and 5%. However, only a minority of these individuals develop the APS. Some estimates indicate that the incidence of APS is around 5 new cases for 100,000 persons for year and the prevalence around 40–50 cases for 100,000 persons [7].

Although the ontogeny of these pathogenic antibodies has not been fully elucidated, some evidences suggests the involvement of both genetic and environmental factors. The ability of aPL to induce a procoagulant phenotype in APS patients plays a central role in the development of arterial and venous thrombotic manifestations typical of the disease. Inflammation serves as a necessary link between this procoagulant phenotype and actual thrombus development. Recent evidence indicated a role for abnormal cellular proliferation and differentiation in the pathophysiology of APS, especially in those patients with pregnancy morbidity and other more atypical manifestations that have no identifiable thrombotic cause [8].

The APS can be found in patients with no clinical or laboratory evidence of another definable condition (“primary” APS), or it may be associated with other diseases (“secondary” APS).

Single-vessel involvement or multiple vascular occlusions may give rise to a wide variety of presentations. Any combination of vascular occlusive events may occur in the same individual, and the time interval between them also varies considerably from weeks to months or even years.

Here, we describe a case of a young man who was diagnosed antiphospholipid syndrome with early complex and unusual manifestations.

## Case presentation

A 43 years old man was admitted to our Internal Medicine Unit on April 2018 with a 7 days history of low-grade fever, bilateral epistaxis and abdominal pain.

His personal medical history showed that 9 years earlier he was hospitalized for subjective dizziness and syncope. Brain MRI showed multiple bilateral posterior parietal hyperintense gliotic lesions, while transthoracic echocardiogram revealed a free floating mass attached to the cusp of the mitral valve with moderate mitral regurgitation. A subsequent transesophageal echocardiogram confirmed the presence of vegetation on mitral valve; a mechanical valve replacement was then performed and a long-term anticoagulant therapy with Acenocoumarol was started with INR target between 2.5–3.5 and TTR target of 70%. The histological analysis of the native valve showed a non-bacterial thrombotic endocarditis. During the recovery, a thrombocytopenia (97,000/mm^3^—n.v. 179–373) was found but no other diagnostic investigation was carried out.

At the age of 41 years, he was admitted to a Neurosurgery Unit for generalized seizures and subsequent head injury. Brain computed tomography (CT) showed a right subdural hematoma and electroencephalography (EEG) background activity was rather irregular, but did not reveal any epileptic abnormalities during wakefulness. The patient was only managed with antiepileptic drugs (phenobarbital). During the hospitalization, a low count of platelet (57,000/mm^3^) was observed and, after haematological evaluation, immune thrombocytopenia (ITP) was diagnosed and steroid therapy (methylprednisolone) was started and extended for 10 days with satisfactory clinical outcome. During the haematological follow-up, courses of therapy with methylprednisolone lasting 7–10 days were administered, taking into account the platelet count.

He had a family history of diabetes and hypertension, he was allergic to aspirin and at admission to our clinical unit he was taking therapy with ramipril, carvedilol, phenobarbital and acenocumarol.

At admission to our clinical unit the physical examination revealed a diffused reduction of vesicular murmur with right basal crackles, ache and pain in the upper abdominal quadrants and splenomegaly.

Laboratory findings were as follows: platelets (PLT) 25 × 10^3^/mm^3^ (n.v. 179–373), prothrombin time (PT)—INR 4,58 (n.v. < 1.2), activated Partial Thromboplastin Time (aPTT) ratio 2.54 (n.v. < 1.20), Amylase 104 U/L (n.v. 8–53), Lipase 1399 U/L (n.v. 73–393), gamma glutamyl transferase (GGT) 393 mU/mL (n.v. 15–85), C-Reactive-Protein (CRP) 149 mg/L (n.v. < 2.9), Lactate Dehydrogenase (LDH) 333 mU/mL (n.v. 87–241); haemoglobin, white blood cell (WBC), serum urea, creatinine, electrolytes, glycaemia, alanine aminotransferase (ALT), aspartate aminotransferase (AST) and bilirubin levels were all within the normal range. Microbiological tests (i.e. Toxoplasma, CMV, HSV, EBV, HIV, HBV, HCV) were all negative and neoplastic markers were within the normal values. Peripheral blood smear showed only megaplatelets.

Workup for autoimmune disorder revealed high titre of anti-β2 glycoprotein I (β2GPI) IgG (113.4 U/mL) and anti-Cardiolipin (aCL) IgG (> 640 U/mL) antibodies, and a low titre of anti-β2GPI and anti-aCL IgM (26.7 U/mL and 24.3 U/mL, respectively); whereas, antiplatelets antibody, Antinuclear antibody, anti ds-DNA, Anti-Mitochondrial antibody (AMA), Antineutrophil cytoplasmic antibody (ANCA), Anti-Liver-Kidney Microsomal Antibody (LKM) and Anti-Smooth Muscle Antibody (ASMA) were negative.

Lupus Anticoagulant (LAC) was performed using Silica Clotting Time (SCT) and Diluted Russell Viper Venom Time (dRVVT) test in three steps (screening, mixing and confirmatory); positive results confirmed the presence of inhibitors of hemostasis, although false positive test may result from interference by concurrent anticoagulant therapy. Anyway, given the presence of both aCL and a β2GPI antibodies, the test was not retested during anticoagulation suspension.

The ultrasound abdomen exam was normal, except of splenomegaly (14 cm). Total body CT confirmed splenomegaly and showed right lower and middle lobe pneumonia; pelvic and other abdominal organs were normal.

Based on the multiple previous courses of therapy with steroid that may facilitate pseudomonas lung infection, a dual antibiotic therapy with piperacillin/tazobactam and levofloxacin was given with sudsequent pneumonia resolution.

The anticoagulation therapy was carried on and steroid therapy with methylprednisolone was set up with increase of platelet count (61 × 10^3^/mm^3^). Supported therapy for the pancreatic insult was also administrated with subsequent normalization of pancreatic enzymes.

On the basis of medical history, clinical and laboratory features, despite the lack of confirmed clinical criteria, and ruling out associated autoimmune diseases, diagnosis of primary antiphospholipid syndrome was made.

At 3 month follow-up, the patients was free of symptoms; CRP, LDH, GGT were restored at normal range; platelet count was 57 × 10^3^/mm^3^; the presence of Anti-*β*2GPI antibodies IgG (63.7 U/mL—middle titre) and IgM (20.5 U/mL—low titre), aCL antibodies IgG (> 640 U/mL—high titre) and IgM (26.7 U/mL—low titre) was confirmed.

## Discussion and conclusions

Antiphospholipid syndrome (APS) is an autoimmune disease characterized by the occurrence of venous and/or arterial thrombosis, and the detection of circulating antiphospholipid antibodies [9].

Single or multiple thrombi in veins, arteries and the microvasculature may give rise a wide range of clinical pictures. While deep vein thrombosis, particularly of the lower limbs, is the most frequently reported clinical manifestation (39%), thrombocytopenia (30%), livedo reticularis (24%), stroke (20%), pulmonary embolism (14%), heart valve lesions (10%), epilepsy (7%), myocardial infarction (6%), leg ulcers (5%) and amaurosisfugax (5%) may also occur and they are classified as “non-criteria features of APS” [10].

The average age of primary APS patients has been reported to be about 35–40 years and the disease is more common in women than in men.

In this report we described a rare case of an adult male who presented over a period of 9 years with a wide spectrum of clinical manifestations involving different organs that were not initially diagnosed as APS.

Dizziness and syncope were his first clinical symptoms, and a non-bacterial thrombotic endocarditis (NBTE) involving the mitral valve was at first diagnosed. Such as, a mechanical valve replacement and oral anticoagulant therapy were carried out.

In APS, heart valve disease occurs in approximately 14% of the patients [11] as valve masses (nonbacterial vegetations) or thickening [12]. The pathogenesis of NBTE in APS has generally been assumed to involve the formation of fibrin-platelet thrombi on the altered valve, with subsequent valve fibrosis, distortion, and finally dysfunction. It has been supposed that antiphospholipid antibodies (aPL) mediate valvular damage merely by promoting thrombus formation on the injured valve endothelium rather than playing a more direct pathogenetic role [13]. The mitral valve is mainly affected, followed by the aortic valve, and the predominant functional abnormality is regurgitation, while stenosis is rare. The indications for surgery are similar to infective endocarditis [14, 15]. NBTE associated to APS may present with nonspecific symptoms which may confuse the further etiology investigations; in addition, the urgent need of surgery may shift the focus to prevention and management of its complications.

At the same time, the brain MRI showed multiple hyperintense gliotic lesions which may be compatible with a demyelination process due to an immune aetiology, based on aPL-brain phospholipid interaction. However, they also can be related to thrombotic events and be the expression of transitory circulation disorder.

In the present case, it could be supposed a cardioembolic etiology of the lesions related to the NBTE. It has also to be stressed that, because of their aspect and localization, the gliotic lesions did not specifically account for syncope and dizziness.

Furthermore, in the first hospitalization mild thrombocytopenia was also reported which got worse in the next hospitalization, when a diagnosis of immune thrombocytopenia was performed and a cycle of steroid therapy was started and resulted in a satisfactory clinical outcome. In this second hospitalization, the patient also presented with generalized seizures and subsequent head injury; brain CT showed a right subdural hematoma while EEG did not reveal any epileptic abnormalities during wakefulness; the patient started taking antiepileptic therapy with gradual improvement.

It has been reported that the prevalence rate of epilepsy and seizures as neurological manifestations of APS is nearly 10 times higher than that in the general population [16]. Evidence suggests that 20% of idiopathic juvenile epilepsy cases are associated with aPL. The pathogenesis is complex and may involve micro-thrombosis, as well as a possible immune-mediated neuronal damage. A proposed mechanism of neuronal injury focuses on the disruption of the blood–brain barrier, secondary to diffuse endothelial dysfunction caused by aPL binding [17]. Furthermore, it has been shown that aPL can impair GABA receptor activity and induce depolarization of synaptoneurosomes, disrupting neuronal function by acting on nerve terminals [18].

When the patient was admitted to our clinic, 2 years after his second hospitalization, with bilateral epistaxis and fever, thrombocytopenia was revealed once more with PT INR increase likely related to anticoagulant-therapy. Moreover, laboratory examinations showed acute pancreatitis with an increase of levels of inflammation markers, while TC revealed right lower and middle lobe pneumonia and a mild splenomegaly.

Based on the patient’s medical history and all the examination results, an autoimmune disorder was supposed and the autommunity workup showed the presence of β2GPI antibodies, aCL antibodies and LAC. As such, despite the lack of confirmed clinical “criteria” of APS, due to the presence of multiple specific “non-criteria features of APS”, namely thrombocytopenia, neurological manifestations, non-bacterial thrombotic endocarditis, pancreatitis, along with the persistence of a high titre of aPL 3 months apart, and given the absence of associated autoimmune diseases, a diagnosis of primary APS was made.

Thrombocytopenia, as a manifestation of primary APS, has a prevalence approximately of 20 to 40% [19]. Antiphospholipid antibodies have been suggested to bind to the phospholipids of the platelet membrane, thus participating to the process of platelet destruction, ultimately leading to thrombocytopenia. However, a clear demonstration of such a role for aPL has never been given. Immunosuppressive therapy appears to be efficacious in patients with APS-associated thrombocytopenia [13]; indeed, a short cycle of intravenous prednisolone resulted in increasing in platelet count in our patient.

Although initial findings of thrombocytopenia and a prolonged aPTT were indicative of a bleeding rather than a prothrombotic disorder, LAC positivity, and, to a less extent, aCL antibody positivity, were associated with an enhanced risk of thrombosis.

We assumed that the pancreatic injury, in the absence of another clear cause, would be due to thrombosis of pancreatic blood vessels. In literature there are a small number of cases of pancreatitis in association with APS [20]. The pathogenesis of this abnormality is not yet clear; however, preliminary autopsy reports suggest a thrombotic rather than an inflammatory etiology [21, 22].

Finally, our patient was admitted to our division with pneumonia. Many infections may be accompanied by increases in aPL and, in some cases, these increases may be associated with clinical manifestations of the APS [23]. Particularly, skin infections (18%), HIV infection (17%), pneumonia (14%), HCV (13%), and urinary tract infections constituted the most common infections found as “triggering” factors for APS [24]. However, the persistent high titre of aPL, after 12 weeks from first referral, permitted us to exclude a transient antibody increase and thus to ascertain diagnosis of APS.

This case represented a challenge in diagnostic process.

It can be very difficult to diagnose at times an APS at the beginning in the absence of classical symptoms.

In the present case, starting from diagnosis of thrombocytopenia, we retraced the patient’s medical history and, after laboratory data confirmation, we were allowed to conclude that all of manifestation were attributable to APS. Indeed, sequential appearance of different and atypical features over a 9-year-long period hampered a prompt diagnostic assessment, until the various clinical manifestations were finally interpreted as epi-phenomena of a unique clinical entity, rather than unrelated diseases.

Though APS is one of the most common thrombocytophilias, unfortunately, it is not recognized often enough. The lack of prevention in undiagnosed patients may cause severe complications such as cerebral stroke, intracerebral haemorrhage, valve damage or myocardial ischemic events, pulmonary emboli and infarction, acute renal failure and hypertension, severe infections which can in turn result in the death of those patients.

## References

[1] Khamashta MA, Bertolaccini Ml, Hughes GR (2004). Antiphospholipid (Hughes) syndrome. Autoimmunity.

[2] Ortel TL (2012). Antiphospholipid syndrome laboratory testing and diagnostic strategies. Am J Hematol.

[3] Chighizola CB, Ubiali T, Meroni PL (2015). Treatment of thrombotic antiphospholipid syndrome: the rationale of current management—an insight into future approaches. J Immunol Res.

[4] Ahluwalia Jasmina, Sreedharanunni Sreejesh (2017). The laboratory diagnosis of the antiphospholipid syndrome. Indian J Hematol Blood Transfus..

[5] Devreese KMJ, Ortel TL, Pengo V, De Laat B (2018). Laboratory criteria for antiphospholipid syndrome: communication from the Ssc of the Isth. J Thromb Haemos.

[6] Garcia D, Erkan D (2018). Diagnosis and management of the antiphospholipid syndrome. N Eng J Med..

[7] Petri M (2000). Epidemiology of the antiphospholipid antibody syndrome. J Autoimmun.

[8] Willis R, Pierangeli SS (2011). Pathophysiology of the antiphospholipid antibody syndrome. Auto Immun Highlights..

[9] Forastiero R (2012). Bleeding in the antiphospholipid syndrome. Hematology..

[10] Atanassova Penka A (2007). antiphospholipid syndrome and vascular ischemic (occlusive) diseases: an overview. YonseiMed J..

[11] Cervera R, Piette JC, Font J, Khamashta MA, Shoenfeld Y, Camps MT (2002). Antiphospholipid syndrome: clinical and immunologic manifestations and patterns of disease expression in a cohort of 1000 patients. Arthr Rheumatol.

[12] Kolitz T, Shiber S, Sharabi I, Winder A, Zandman-Goddard G (2019). Cardiac Manifestations of Antiphospholipid Syndrome With Focus on Its Primary Form. Front Immunol..

[13] Ibrahim AM, Siddique MS (2019). Libman Sacks Endocarditis. StatPearls.

[14] Asopa S, Patel A, Khan OA, Sharma R, Ohri SK (2007). Non-bacterial thrombotic endocarditis. Eur J Cardiothorac Surg.

[15] Rabinstein AA, Giovanelli C, Romano JG, Koch S, Forteza AM, Ricci M (2005). Surgical treatment of nonbacterial thrombotic endocarditis presenting with stroke. J Neurol.

[16] Noureldine MH, Harifi G, Berjawi A, Haydar AA, Nader M, Elnawar R, Sweid A, Al Saleh J, Khamashta MA, Uthman I (2016). Hughes syndrome and epilepsy: when to test for antiphospholipid antibodies?. Lupus..

[17] Katzav A, Shoenfeld Y, Chapman J (2010). The pathogenesis of neural injury in animal models of the antiphospholipid syndrome. Clin Rev Allergy Immunol.

[18] Chapman J, Cohen-Armon M, Shoenfeld Y, Korczyn AD (1999). Antiphospholipid antibodies permeabilize and depolarize brain synaptoneurosomes. Lupus.

[19] Diz-Kucukkaya R, Hacihanefioglu A, Yenerel M (2001). Antiphospholipid antibodies and antiphospholipid syndrome in patients presenting with immune thrombocytopenic purpura: a prospective cohort study. Blood.

[20] Spencer HL (2004). Primary antiphospholipid syndrome as a new cause of autoimmune pancreatitis. Gut.

[21] Uthman I, Khamashta M (2007). The abdominal manifestations of the antiphospholipid syndrome. Rheumatology (Oxford).

[22] Asherson RA, Cervera R (2003). Unusual manifestations of the antiphospholipid syndrome. Clin Rev Allergy Immunol.

[23] Asherson R, Cervera R (2003). Antiphospholipid antibodies and infections. AnnRheumDis..

[24] Cervera R, Asherson RA (2005). Antiphospholipid syndrome associated with infections: clinical and microbiological characteristics. Immunobiology..

